# Projecting Health Impacts of Future Temperature: A Comparison of Quantile-Mapping Bias-Correction Methods

**DOI:** 10.3390/ijerph18041992

**Published:** 2021-02-18

**Authors:** Weijia Qian, Howard H. Chang

**Affiliations:** Department of Biostatistics and Bioinformatics, Emory University, Atlanta, GA 30322, USA; weijia.qian@emory.edu

**Keywords:** health impact, climate change, temperature, emergency department visits, bias-correction, quantile mapping

## Abstract

Health impact assessments of future environmental exposures are routinely conducted to quantify population burdens associated with the changing climate. It is well-recognized that simulations from climate models need to be bias-corrected against observations to estimate future exposures. Quantile mapping (QM) is a technique that has gained popularity in climate science because of its focus on bias-correcting the entire exposure distribution. Even though improved bias-correction at the extreme tails of exposure may be particularly important for estimating health burdens, the application of QM in health impact projection has been limited. In this paper we describe and apply five QM methods to estimate excess emergency department (ED) visits due to projected changes in warm-season minimum temperature in Atlanta, USA. We utilized temperature projections from an ensemble of regional climate models in the North American-Coordinated Regional Climate Downscaling Experiment (NA-CORDEX). Across QM methods, we estimated consistent increase in ED visits across climate model ensemble under RCP 8.5 during the period 2050 to 2099. We found that QM methods can significantly reduce between-model variation in health impact projections (50–70% decreases in between-model standard deviation). Particularly, the quantile delta mapping approach had the largest reduction and is recommended also because of its ability to preserve model-projected absolute temporal changes in quantiles.

## 1. Introduction

Global warming affects human health by increasing population exposures to hazardous environmental conditions, such as prolonged high temperature, elevated ambient air pollution, and extreme weather events (e.g., floods, hurricanes and dust storms) [1,2]. Evidence supporting historical and future warming of the climate system is unequivocal; however, the magnitude and where changes will have the most impact are still uncertain. Estimates of future health burdens attributable to environmental risks under different emission scenarios can help quantify the significance of climate change, as well as provide crucial information for decision makers in developing long-term strategies to protect public health and maintain environmental sustainability [3].

Health-impact projection is conducted by combining estimated health-exposure relationships with projected future exposures while considering various sources of uncertainties in the analysis [4]. Future exposures are typically derived using simulations from computationally expensive climate models. Even though these models reflect state-of-the-art knowledge on the climate system, their outputs are known to exhibit complex spatial–temporal biases when compared to observations. Factors contributing to this bias include errors in parameters describing physical and chemical processes, incorrect representation of the underlying processes with mathematical equations, and discretization of meteorological fields in space and in time. Hence, climate-model simulations for the projection period need to be bias-corrected prior to estimating future health impacts.

In order to assess temporal changes in meteorological trends, climate model simulations are performed for both historical (hindcast) and future (projection) periods. Generally, bias-correction for future simulation is accomplished in two steps. First, the bias between observations and simulations during the hindcast period is assessed. Then a correction algorithm is applied to future simulations by assuming the bias can be extrapolated to future periods. In a recent systematic literature review of heat-related mortality projections [5], among the 63 identified studies, 59 studies applied methods to address bias in climate model outputs. However, the most commonly used bias-correction methods were based on shifting or scaling climate model simulations that have been shown to perform poorly for removing bias at extremes [6].

Quantile mapping (QM) is a recent bias-correction approach that has gained popularity in climate science [7]. It is motivated by the need to characterize biases over the entire distribution of the climate variable. Several studies have found that QM outperforms other simpler methods (i.e., shifting or scaling) for temperature and precipitation for different statistical characteristics of interest (e.g., standard deviation, percentiles) [8,9,10,11,12]. More importantly, QM has also been shown to reduce variation between different climate models, hence reducing a well-recognized source of projection variability. The ability to bias-correct the entire exposure distribution is particularly relevant for calculating health burdens. This is because health effects of environmental exposures are often nonlinear, and extreme exposures are often associated with higher adverse effects. Bias-correction methods that directly target the extreme tails may result in better projections compared to methods that focus on the mean trend in future exposures. However, the use of QM in health-impact projection has been limited but is increasing [13,14,15].

The main objective of this paper is to investigate the use of QM methodologies in conducting health-impact projections. We aim to assess the robustness of health-impact estimates against different QM assumptions and assess the usefulness of QM in reducing uncertainties in a climate model ensemble. Our case study involves estimating future excess emergency department visits attributable to changes in daily warm-season minimum temperature in the Atlanta metropolitan area for the period 2050 to 2099. The focus on minimum temperature is motivated by historical trends of increasing humidity and minimum temperatures in the southeastern region of the United States [16,17]. Minimum temperature also corresponds to night-time temperature that has been associated with increased health risks in Atlanta and other locations [18,19,20,21].

## 2. Materials and Methods

### 2.1. Metrorology and Health Data

We utilized climate model simulations from the North American-Coordinated Regional Climate Downscaling Experiment (NA-CORDEX) [22]. The NA-CORDEX program is designed to evaluate dynamic downscaling methodologies using different combinations of regional climate models (RCM) and global climate models (GCM). This analysis included daily minimum temperature simulations from 10 RCM-GCM models under the Representative Concentration Pathway (RCP) 8.5, which describes a worst-case or business-as-usual future scenario. We linked the approximately 50 km climate model grid cell to the meteorology monitor at the Atlanta Hartsfield-Jackson International Airport, and extracted model hindcast simulations over (1) a 10-year historical period of 1993 to 2004, and (2) a 50-year period of 2050 to 2099.

RCM included the Canadian Regional Climate Model version 4 (CanRCM4), the Canadian Regional Climate Model version 5 by Université du Québec à Montréal (CRCM-UQAM), the High-Resolution Limited Area Model with ECHAM physics, version 5 (HIRHAM5), the Regional Climate Model version 4 (RegCM4), the Rossby Centre regional atmospheric model version 4 (RCA4), and the Weather Research and Forecasting model (WRF). GCM included the second-generation Canadian Earth System Model (CanESM2), the European community Earth-System Model (EC-EARTH), the Geophysical Fluid Dynamics Laboratory Earth System Models (GFDL-ESM2M), the Met Office Hadley Centre with the HadGEM2-ES Earth System model (HadGEM2-ES), and the coupled Max Planck Institute Earth System Model with mixed resolution (MPI-ESM-MR) and low resolution (MPI-ESM-LR). Specific RCM-GCM model combinations are given in Table 1.

Daily counts of emergency department (ED) visits for the 20-county Atlanta metropolitan area were obtained by aggregating patient-level records from individual hospitals and the Georgia Hospital Associations for the same historical period 1993 to 2004. We used the International Classification of Disease version 9 (ICD-9) codes to identify ED visits for all internal causes (ICD-9: 001–799) in the primary or secondary diagnosis fields. We restricted the analysis to warm months of May to September. The total number of ED visits was 6,994,110 with an average of 2286 per day.

### 2.2. Bias Correction with Quantile Mapping

We first describe the general approach of QM. Let $x_{o,h}\left(t\right)\;$denote the *observation* at time *t* during the *historical* period with cumulative distribution function (CDF) defined as $F_{o,h}\left(z\right)=P\;\left[x_{o,h}\left(t\right)\leq z\right]$. Specifically, $F_{o,h}\left(z\right)$ gives the probability that an observed historical temperature will be less than *z*. For a quantile level *τ*, 0 ≤ *τ* ≤ 1 the quantile value (also known as the τ-th percentile) is obtained by the inverse CDF: $F_{o,h}^{-1}\left(\tau \right)$. Also let $x_{m,h}\left(t\right)\;$and $x_{m,p}\left(t\right)\;$denote, respectively, climate *model data* during the *historical* and the *projected* future periods, with corresponding CDFs $F_{m,h}\left(z\right)$ and $F_{m,p}\left(z\right)$.

Let ${\hat{x}}_{o,p}\left(t\right)$ denote the estimated future observation at time *t* during the projection period. This is obtained by *bias-correcting* future model simulation via a transfer function $g\left(\;\right)$ such that ${\hat{x}}_{o,p}\left(t\right)=g\left[x_{m,p}\left(t\right)\right]$. Different QM methods derive $g\left(\;\right)$ by using the CDFs or quantile functions of observed and model data during an overlapping historical period. Below we describe the five QM methods considered in this analysis. Example R code for implementation is provided in Supplementary Materials.

#### 2.2.1. Normal Distribution Mapping

Assuming the outcome follows a Normal distribution, this approach utilizes the theoretical quantile function to perform bias-correction [23,24]. First, Normal distribution parameters (i.e., mean and variance) are estimated separately for the observed $x_{o,h}\left(t\right)\;$and modeled $x_{m,h}\left(t\right)$ data during the historical period. The bias-corrected future projection at time *t* is given by ${\hat{x}}_{o,p}\left(t\right)=F_{o,h}^{-1}\left[F_{m,p}\left(x_{m,p}\left(t\right)\right)\right]$. Here the transfer function $g\left(\;\right)=\;F_{o,h}^{-1}F_{m,p}\left(\;\right)$ first identifies the quantile level $\hat{\tau }$ of a future model value with respect to the model’s historical distribution; then, the corresponding quantile of observed historical distribution is defined as the bias-corrected projection value.

#### 2.2.2. Empirical Quantile Mapping

Empirical QM is a nonparametric method that relaxes the Normal assumption [25]. Specifically, CDFs of historical observation and model data are first estimated over a set of regularly spaced quantile levels, τ=0, 0.01, 0.02, …, 0.99, 1.00. Linear interpolation is then applied to obtain quantile values for levels that are not in the above list [26]. The bias-corrected future projection at time *t* via quantile mapping is given by ${\hat{x}}_{o,p}\left(t\right)=F_{o,h}^{-1}\left[F_{m,p}\left(x_{m,p}\left(t\right)\right)\right]$.

#### 2.2.3. Empirical Robust Quantile Mapping

Robust QM extends the empirical QM method by using nonlinear local linear least squares (NLLS) regression to estimate the quantile–quantile relation of the historical observed and modeled time series [26]. For each quantile level *τ* = 0, 0.01, 0.02, …, 0.99, 1.00, NLLS is applied to estimate a flexible quantile mapping function $g\left(\;\right)$, such that $F_{o,h}^{-1}\left(\tau \right)=g\left[F_{m,h}^{-1}\left(\tau \right)\right]$ using the 10 nearest data points identified in the quantile–quantile plot. Linear interpolation is applied to obtain quantile values for quantile levels that are not in the above set [27]. The above estimation procedure is replicated for 10 bootstrap samples, and the mean of the bootstrap replicates $\bar{g}\left(\;\right)\;$is used as the final mapping function. The bias-corrected future projection at time t is ${\hat{x}}_{o,p}\left(t\right)=$ $\bar{g\;}\left[x_{m,p}\left(t\right)\right]$.

#### 2.2.4. Quantile Mapping with Linear Transformation Function

Linear QM that assumes a linear relationship between quantile functions of the observed and model time series during the historical period: $F_{o,h}^{-1}\left(\tau \right)=a+b\times F_{o,m}^{-1}\left(\tau \right)$. Coefficients *a* and *b* are obtained from fitting a linear least squares regression. The bias-corrected future projection at time *t* is then given by ${\hat{x}}_{o,p}\left(t\right)=a+b\times x_{m,p}\left(t\right)$ [23].

#### 2.2.5. Quantile Delta Mapping (QDM)

QDM aims to preserve the model-projected absolute changes in quantiles following bias correction by QM [28]. Hence, the transfer function is allowed to be time-varying. For model data, the absolute change in quantiles between the historical and future time *t* is $\Delta_{m}\left(t\right)=x_{m,p}\left(t\right)-F_{m,h}^{-1}\left[F_{m,p}\left(x_{\Delta m,p}\left(t\right)\right)\right]$. This change is used to adjust bias-corrected values from the empirical QM results. Specifically, the bias-corrected future projection at time *t* via quantile mapping is given by ${\hat{x}}_{o,p}\left(t\right)=F_{o,h}^{-1}\left[F_{m,p}\left(x_{m,p}\left(t\right)\right)\right]+\;\Delta_{m}\left(t\right)$.

#### 2.2.6. Application to NA-CORDEX and Evaluations

To minimize the impact of seasonality, for each NA-CORDEX RCM/GCM combination, we applied the five QM methods by season (December–February, March–May, June–August, September–November) using the R packages qmap and MBC (for QDM) (R Core Team, Vienna, Austria). To evaluate the model fit different quantile mapping methods on bias-correcting simulation data, we calculated the mean bias error (MBE), mean absolute error (MAE), root mean square error (RMSE), and normalized standard deviation (NSD) between the raw and bias-corrected model values during the historical period. Let ${\hat{x}}_{m,h}\left(t\right)=g\left[x_{m,h}\left(t\right)\right],\;$three statistics are defined as:

(1) $$MBE=\frac{\sum_{t=1}^{n}x_{o,h}\left(t\right)-{\hat{x}}_{m,h}\left(t\right)\;}{n}$$

(2) $$MAE=\;\frac{\sum_{t=1}^{n}{\left|x_{o,h}\left(t\right)-{\hat{x}}_{m,h}\left(t\right)\right|}^{}\;}{n}$$

(3) $$RMSE=\;\sqrt{\frac{\sum_{t=1}^{n}{\left[x_{o,h}\left(t\right)-{\hat{x}}_{m,h}\left(t\right)\right]}^{2}\;}{n}}$$

(4) $$NSD=\;\frac{\sigma^{m}}{\sigma^{o}}$$

where *σ^m^* and *σ^0^* are the standard deviation of the modeled and observed datasets, respectively.

We note that the above metrics are in-sample evaluation of model fit. Out-of-sample evaluations are challenging because climate model projections represent simulations under different emission assumptions.

### 2.3. Health Effect Estimation and Projection

We used quasi-Poisson log–linear model to estimate the association between temperature and daily ED visit counts. Let *μ* be the mean ED counts on day *t*. The time-series model is given by:

(5) $${\log\mu }_{t}=\alpha_{0}+f\left(x_{t}\right)+h\left(t\right)+\;\mathrm{other}\;\mathrm{confounders}$$

where $x_{t}$ is the 3-day moving average of temperature exposure on day t. The above model assumes that the effect of $x_{t}$ is exerted over a 3-day period (same-day, lag 1 and lag 2). We modeled the nonlinear effect of temperature $f\left(x_{t}\right)$ using natural cubic splines with 3 degrees of freedom. Long-term and seasonal trends in the ED visit time series are controlled by $h\left(t\right)$, which was modeled using natural cubic splines with monthly knots. Finally, other confounders in the model included indicators for day-of-the-week, nonlinear effect of dew-point temperature as a measure of humidity, indicators for federal and state holidays, and hospital-specific indicators to account for hospitals’ contributions to the total ED visits in the city. We considered sensitivity of the health model specification on health projection by altering the degrees of freedom for $f\left(x_{t}\right)\;$to 2 and 4, and the number of knots for$\;h\left(t\right)$ to 4, 5, or 7.

For each day *t* during the *historical* period (May–September, 1993 to 2004), the attributable number (AN) associated with temperature exposure is given by:

(6) $$AN_{t,H}=n_{t}\left[1-e^{-\theta_{x}\left(t\right)}\right]$$

where $n_{t}$ is the daily total number of observed ED visits and $\theta_{x}\left(t\right)$ is the log relative risk associated with exposure. Specifically, $\theta_{x}\left(t\right)=f\left(x_{t}\right)-f\left(x_{0}\right)$, where $f\left(\cdot \right)$ is the nonlinear exposure-response function and $x_{0}$ is a reference (baseline) temperature. We defined the reference as the minimum observed exposure (i.e., 7.07 °C) because we found the health association to be strictly increasing. When the association is U-shaped, the temperature with the minimal risk is typically used as the reference.

For projected future ED visits, we used future temperature as counterfactual exposures. For each day *t* during the projection period (May–September, 2050 to 2099), the AN is:

(7) $$AN_{t,P}=\left(n_{t}-AN_{t,H}\right)\times \left[e^{\theta_{{\hat{x}}_{p}}\left(t\right)}-1\right]$$

where $\left(n_{t}-AN_{t,H}\right)$ represents the baseline ED-visits, $\theta_{{\hat{x}}_{p}}\left(t\right)=f\left(\hat{y}\right)-f\left(x_{0}\right)$ and ${\hat{x}}_{o,p}\left(t\right)$ is the bias-corrected projected future temperature. Finally, changes in ED visits due to future increases in exposure can be calculated by aggregating or averaging *AN_t,H_* and *AN_t,P_* over the desired comparison time periods.

Calculations of *AN_t,H_* and *AN_t,P_* involved nonlinear functions of the estimated log relative risk. We obtained point-projection and projection uncertainty intervals via Monte Carlo simulations. Specifically, we first simulated 5000 realizations of the exposure-response function $f\left(\cdot \right)$ by simulating its spline coefficients from a multivariate Normal distribution with their point estimate as means and the asymptotic covariance matrix. These simulations were then combined with projected future temperature time series to perform uncertainty quantification. We report the median as the point projection estimate, and 95% uncertainty intervals were based on the 2.5th and 97.5th quantile of the simulated health-impact projections.

In addition to evaluating health-impact projections by individual climate model, we further considered two ensemble methods for combing projections across models. In the first method, we calculated point and interval projections using the average health projections across the 10 models for each Monte Carlo realization. In the second method, we obtained point and interval projection by pooling all Monte Carlo realizations from individual models. The first method assumes the average projection as the ensemble estimate, while the second approach accounts for between-model variability.

## 3. Results

Table 1 describes differences in raw climate-model simulations and airport observations for minimum temperature over the historical period of 1993 to 2004. Overall, six out of the 10 GCM/RCM combinations had average simulations lower than observations, with an across-model average negative bias of −0.79 °C. Eight out of the 10 models have NSD greater than 1, indicating that these modeled temperatures have higher variability than the observed temperatures. Table 2 summarizes the effectiveness of bias correction for different quantile mapping methods when applied to the historical period. All quantile mapping methods were able to reduce MBE to nearly zero and NSD to 1. QDM, followed closely by Linear QM, had the lowest MBE. QDM and Linear QM are more flexible than the other methods that either impose a distributional assumption (Normal Mapping) or use the empirical quantile function directly without additional transformation.

**Figure 1:**
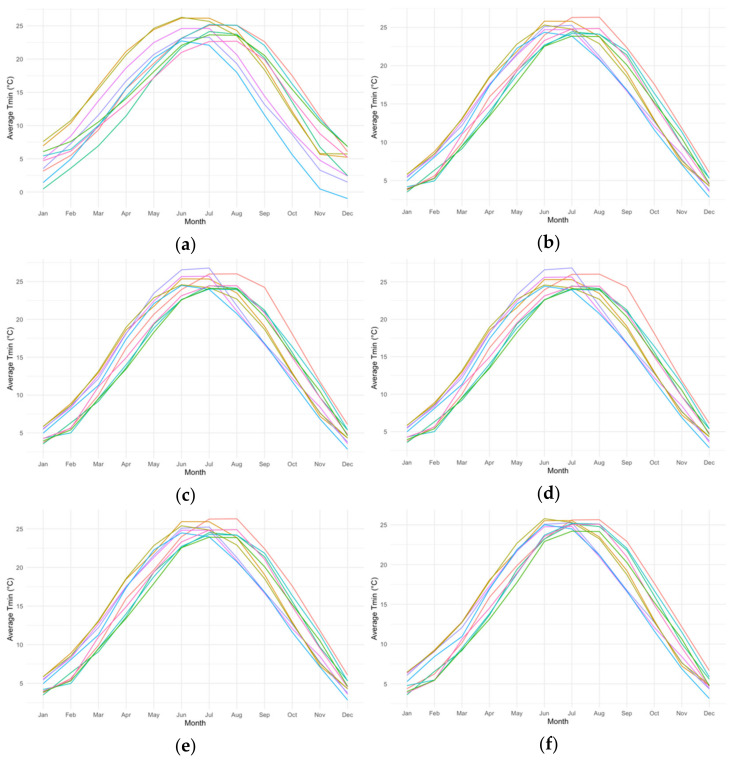
Monthly average minimum temperature in Atlanta (2051–2099) for 10 climate models with and without different quantile mapping bias-correction methods. Each color indicates a different GCM and RCM combination from NA-CORDEX. (**a**) Future monthly mean without correction; (**b**) Future monthly mean by linear QM; (**c**) Future monthly mean by empirical QM; (**d**) Future monthly mean by robust QM; (**e**) Future monthly mean by normal mapping; (**f**) Future monthly mean by QDM.

Figure 1 shows monthly mean minimum temperature for the future period (2050–2099) for different GCM/RCM combinations with and without bias correction. Overall, for most models, applying quantile mappings increased the monthly projected means. This is consistent with the results that the raw simulation tended to underestimate daily minimum temperature during the historical period. Comparing results from different QM methods within each climate model, (shown in Supplementary Figure S1), QDM tended to give the highest projected temperature values. This may be due to QDM’s ability to incorporate projected temporal changes in quantiles and hence extremes values are better preserved. We also observed that application of different QM methods resulted in a more pronounced reduction in between-model variation for the winter months.

More importantly, applying quantile mappings reduced between-model variation. Table 3 gives the average between-model standard deviation for daily May to September temperature during two projection periods (coefficient of variations given in Supplementary Table S1). For the period 2050–2059, raw simulations had a between-model standard deviation of 1.51, while all QM methods reduced the standard deviation to less than 0.7. Similar reductions in between-model variation in future projects are seen in future monthly 95th quantile value (Supplementary Figure S1). Finally, Table 3 also shows increasing between-model variation in the projected temperature further into the future as time moves from the 2050s to 2090s.

**Figure 2:**
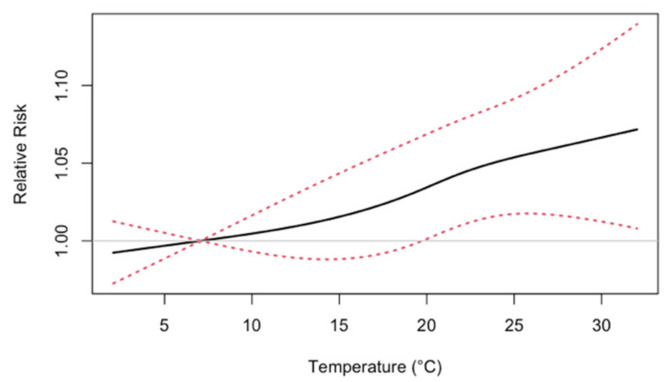
Estimated nonlinear associations between 3-day moving averages of minimum temperature on all internal-cause emergency department visits in Atlanta, 1993 to 2004. The exposure-response function has a reference temperature of 7.07 °C, and dotted lines denote the 95% pointwise confidence interval bounds.

**Figure 3:**
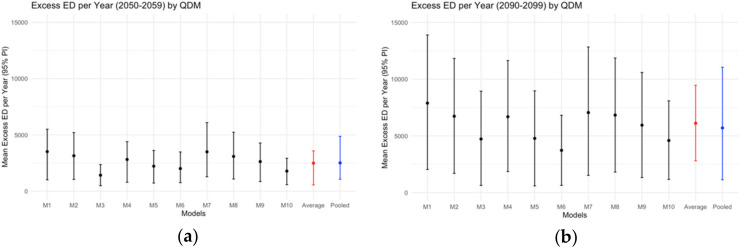
Projected excess temperature-related ED visits per year and projecquantile delta mapping. Specific global-climate model and regional-climate model combinations from NA-CORDEX are given as M1, M2, …, M10 in Table 1. (**a**) Excess ED per year (2050–2059); (**b**) Excess ED per year (2090–2099).

Figure 2 gives the estimated nonlinear associations between 3-day moving averages and ED visits. The estimated association appears to be monotonically increasing with the minimum observed temperature (reference temperature x_0_) at 7.07 °C. Figure 3 summarizes excess temperature-related ED visits in the 2050s and 2090s, after applying bias correction with QDM. The number of excess temperature-related ED visits in the 2090s are projected to be higher than that in the 2050s within the same RCM/GCM combination. The projection uncertainty also increased from the 2050s to 2090s. Based on 5000 Monte Carlo simulations from the 10 RCM/GCM combinations, the pooled ensemble approach projected excess temperature-related ED visits per year as 2510 (95% PI: 700–5000) in the 2050s and 5900 (95% PI: 1000–11700) in the 2090s. Without incorporating between-model variability, the average ensemble approach gave similar point projection but slightly small projection intervals. In the sensitivity analysis of alternative health models (Table S2), the pooled projections of annual excess ED visits are similar and have considerable overlap in projection intervals.

Finally, Table 3 shows that applying quantile mappings reduced the between-model variation in projected annual excess ED visits considerably. For example, QDM reduced the between-model projection standard deviation by 72% for the 2050s and 53% for the 2090s. ED visit projections for all RCM/GCM combinations and bias-correction methods are given in Supplementary Table S2. Overall, we found consistent projections across bias-correction methods with large overlapping prediction intervals.

## 4. Discussion

Quantile mapping (QM) has become widely used in bias-correcting climate-model simulations because of its ability to characterize distribution tails more flexibly. We describe, to the best of our knowledge, the first evaluation of different QM methods in projecting future health impacts associated with temperature. Using an ensemble of regional climate model simulations from the NA-CORDEX experiment, we found consistent increases in ED visits attributable to future changes in daily ambient minimum temperature under the RCP 8.5.

From the recent review of heat-related mortality projections [5], delta change is the most commonly used method to account for climate-model bias. Here, differences in the climate-model simulations between the baseline and projection periods are first calculated. Future projections are then obtained by adding this difference to the observed historical exposures. This method makes the assumption that climate-model bias will cancel out. However, when applied to health-impact projections, the choice of baseline period is challenging because the availability of health data is usually shorter and more recent. Specifically, for our case study, even though NA-CORDEX has a hindcast period of 1950 to 2005, the temperature–health association was estimated only using data from 1993 to 2004.

Several studies have compared results from applying different QM methods to bias-correct meteorology data. Murdock et al. (2015) suggested that traditional QM altered relative trends in precipitation extremes projected by GCMs, while quantile delta mapping (QDM) was able to preserve relative trends [28]. Tong et al. (2020) applied QM and QDM to RegCM4-simulated temperature data and reported that QDM preserved projected changes in temperature well, but QM artificially modified the temperature change signal in both magnitude and pattern [29]. In Enatayi et al. (2020), the empirical QM and robust QM approaches performed best for correcting RCM-simulated rainfall data, while all QM methods, except a parametric QM, performed relatively well for RCM-simulated temperature data [30]. These results are consistent with our health projection analyses using different QM methods.

There are several additional variations of QM approaches that were not included in our analysis. First, smoothing splines (SSPLIN) is a nonparametric QM method in which a smoothing spline is used to fit the quantile–quantile plot of the observed and modeled data [31]. However, Chu at al. (2020) showed that SSPLN performed worse than the other QMs in correcting temperature bias [30]. Detrended quantile mapping (DQM) is designed to preserve projected changes in the modeled mean but does not necessarily preserve changes in all quantiles [28]. Compared to QDM, DQM tended to perform worse in reproducing projected changes marginally [28]. Finally, scaled distribution mapping (SDM) is another method that accounts for climate temporal trends. Unlike QDM, it uses a parametric model instead of a nonparametric one and more explicitly accounts for differences in the modelled variances between the baseline and future period [32].

Besides temperature, QM algorithms are also widely used in bias-correcting other simulated meteorology variables such as precipitation [29,33,34] and ozone density [18]. Applying QM to precipitation data requires special considerations, since climate models typically underestimate the number of dry days due to the drizzle effect [35]. When dry-day correction is needed, an optimal threshold of precipitation intensity needs to be derived, and climate model simulation values below the threshold are set to zero. When using parametric-based QM, unlike temperature, which is well represented by a Gaussian distribution [23], precipitation usually employs a Gamma or mixed distribution [23,30].

Finally, our health-impact projections of future ED visits have several limitations. First, we did not consider changes in population or changes in baseline ED visit rates. Population change is driven by migration, life expectancy and fertility, and these variables are interrelated to anthropogenic emission. Second, future baseline ED visit rates may change in response to the overall health of the population and health care access. Hence, our estimates should be interpreted as a *counterfactual scenario* of future daily minimum temperature time series (e.g., 2051–2060) occurring during the historical period 1993 to 2004. Moreover, changes in the at-risk population and baseline risks will likely have little influence on our comparison of different bias-correction methods because these parameters are not related to future exposure projections. Particularly, information regarding projected changes in population size and baseline ED rate can be incorporated in the health-impact calculations by replacing the parameter *n_t_* with its projected values.

## 5. Conclusions

In this case study of ED visits and daily minimum temperature in Atlanta, we estimated consistent increase in ED visits across climate models under RCP 8.5 during the period 2050 to 2099. We found that QM methods can significantly reduce between-model standard deviation by 50–70% in health projections. Particularly, the quantile delta mapping (QDM) approach is recommended because it gave the largest reduction in between-model variation, it resulted in good model fit during the hindcast historical period, and the approach aims to preserves model-projected absolute temporal changes in quantiles.

## Figures and Tables

**Table 1:** Ten combinations of global climate models (GCM) and regional climate models (RCM) from the North American-Coordinated Regional Climate Downscaling Experiment (NA-CORDEX) used in this study. Mean bias error (MBE), mean absolute error (MAE), root mean square error (RMSE), and normalized standard deviation (NSD) in °C are between model simulations and airport observations during 1993 to 2004.

Model Index	GCM	RCM	MBE	MAE	RMSE	NSD
**M1**	HadGEM2-ES	WRF	−0.55	4.66	6.26	1.06
**M2**	CanESM2	CRCM5-UQAM	0.88	4.68	6.16	1.03
**M3**	MPI-ESM-LR	WRF	−0.88	4.81	6.49	1.10
**M4**	MPI-ESM-LR	RegCM4	−2.22	4.95	6.47	1.10
**M5**	GFDL-ESM2M	WRF	−2.25	4.98	6.53	1.05
**M6**	GFDL-ESM2M	RegCM4	−3.40	5.48	7.11	1.08
**M7**	CanESM2	CanRCM4	0.75	4.71	6.26	1.01
**M8**	MPI-ESM-MR	CRCM5-UQAM	0.18	4.97	6.53	1.01
**M9**	EC-EARTH	RCA4	−0.98	4.67	6.02	0.88
**M10**	EC-EARTH	HIRHAM5	0.60	4.45	5.94	0.92

Canadian Regional Climate Model version 4 (CanRCM4), the Canadian Regional Climate Model version 5 by Université du Québec à Montréal (CRCM-UQAM), the High-Resolution Limited Area Model with ECHAM physics, version 5 (HIRHAM5), the Regional Climate Model version 4 (RegCM4), the Rossby Centre regional atmospheric model version 4 (RCA4), and the Weather Research and Forecasting model (WRF); second-generation Canadian Earth System Model (CanESM2), the European community Earth-System Model (EC-EARTH), the Geophysical Fluid Dynamics Laboratory Earth System Models (GFDL-ESM2M), the Met Office Hadley Centre with the HadGEM2-ES Earth System model (HadGEM2-ES), and the coupled Max Planck Institute Earth System Model with mixed resolution (MPI-ESM-MR) and low resolution (MPI-ESM-LR).

**Table 2:** Comparison of raw and quantile-mapping (QM) bias-corrected climate model simulations. Mean bias error (MBE), mean absolute error (MAE), root mean square error (RMSE), and normalized standard deviation (NSD) in °C were between model simulations and airport observations during 1993 to 2004 and averaged across climate models.

Metric	Raw	Linear QM	Empirical QM	Robust QM	Normal Mapping	QDM
**MBE (** ×10)	−7.86	−0.00	0.02	0.01	0.02	0.00
**MAE**	4.84	4.36	4.35	4.35	4.37	4.35
**RMSE**	6.38	5.78	5.78	5.78	5.80	5.78
**NSD**	1.02	1.00	1.00	1.00	1.00	1.00

**Table 3:** Average between-model standard deviation (SD) for projected daily minimum temperature and excess emergency department (ED) visits in Atlanta between May and September for two projection periods. Statistics were first calculated across days within each climate model and then across 10 models from NA-CORDEX.

Quantile-Mapping Methods	Future Min Temperature (SD Across Climate Models)		Future Excess Ed Visits (SD Across Climate Models)	
	2050–2059	2090–2099	2050–2059	2090–2099
**Raw**	1.51	1.80	2580	2889
**Linear QM**	0.58	0.85	958	1400
**Empirical QM**	0.68	0.82	1139	1341
**Robust QM**	0.68	0.84	1135	1331
**Normal Mapping**	0.58	0.86	970	1424
**QDM**	0.44	0.78	723	1356

## Data Availability

The climate data used in this study can be downloaded from https://na-cordex.org/ (accessed on 12 February 2021). The emergency department visits contain protected health information and cannot be shared due to existing data use agreement.

## Supplementary Materials

The following are available online at https://www.mdpi.com/1660-4601/18/4/1992/s1, Table S1: Average between-model coefficient of variation for projected daily minimum temperature and excess emergency department (ED) visits between May and September for two projection periods. Table S2: Sensitivity of health-impact projections under alternative health model specifications. Figure S1: Monthly average minimum temperature in Atlanta (2051–2099) for 10 climate models with and without different quantile mapping bias-correction methods.
