# Supplementary material for: Projecting Health Impacts of Future Temperature: A Comparison of Quantile-Mapping Bias-Correction Methods
Source: Int J Environ Res Public Health. 2021 Feb 18;18(4):1992. doi: 10.3390/ijerph18041992 (PMC7922393; doi:10.3390/ijerph18041992)
Supplement: Supplementary file 1 [file ijerph-18-01992-s001.pdf]

International Journal of
Environmental Research and Public Health

MDPI

Table S1. Average between-model **coefficient of variation** for projected daily minimum temperature and excess emergency department visits (ED) between May and September for two projection periods. Statistics were first calculated on each day across 10 model simulations from NA-CORDEX and then averaged across days.

	Future Minimum Temperature		Annual Excess ED Visits	
	**2050-2059**	**2090-2099**	**2050-2059**	**2090-2099**
**Raw**	0.07	0.08	1.97	0.63
**Linear QM**	0.03	0.04	0.39	0.25
**Empirical QM**	0.03	0.04	0.41	0.23
**Robust QM**	0.03	0.04	0.41	0.23
**Normal Mapping**	0.03	0.04	0.39	0.25
**QDM**	0.02	0.03	0.28	0.23

Table S2. Sensitivity Analysis by varying the df for temperature and time. Projected excess temperature-related ED visits per year and 95% projection intervals (PI) in the 2050s and 2090s after applying bias-correction with quantile delta mapping. Model for the primary analysis is indicated in bold.

DF_{temp}	DF_{time}	Annual Excess ED Visits (Pooled)	
		**2050-2059**	**2090-2099**
2	4	3000 (900-5500)	7200 (3000-12300)
2	5	2900 (900-5200)	7000 (3000-11900)
2	6	3000 (1100-5300)	7300 (3200-12300)
2	7	3100 (1100-5500)	7600 (3400-12600)
3	4	2800 (700-5500)	6500 (1600-12500)
3	5	2800 (800-5400)	6800 (1900-12800)
**3**	**6**	**2600 (700-5000)**	**5900 (1000-11700)**
3	7	3000 (1000-5600)	7100 (2100-13200)
4	4	3000 (800-6000)	7400 (1800-14300)
4	5	2700 (700-5300)	6100 (700-12800)
4	6	2500 (600-5100)	5500 (100-12000)
4	7	2300 (900-5700)	7000(1300-13700)

Example R Code

```
#### Implementing quantile-mapping bias correction methods

# Obs = observations during historical period
# Mod.Hist = climate model output during historical period
# Mod.Proj = climate model output during projection period.

library('qmap')

### QM1: Linear transform function
qm1.fit <- fitQmap(Obs, Mod.Hist, method = "PTF", transfun = "linear", wet.day =
FALSE, cost = "RSS")
```

```
qm1.proj <- doQmapPTF(Mod.Hist, qm1.fit)
qm1.hist <- doQmapPTF(Mod.Proj, qm1.fit)


### QM2: Empirical quantile mapping
qm2.fit <- fitQmapQUANT(Obs, Mod.Hist, qstep = 0.01, wet.day = FALSE)
qm2.proj <- doQmapQUANT(Mod.Proj[check.na], qm2.fit)
qm2.hist <- doQmapQUANT(Mod.Hist, qm2.fit, qstep = 0.01, wet.day = FALSE)


### QM3: Empirical robust quantile mapping (non-parametric)
qm3.fit <- fitQmap(Obs, Mod.Hist, qstep = 0.01, method = "RQUANT", wet.day = FALSE)
qm3.proj <- doQmap(Mod.Proj, qm3.fit, type = "linear")
qm3.hist <- doQmap(Mod.Hist, qm3.fit, type = "linear")


### QM4: Normal distributional mapping
qm4.fit <- fitQmap(Obs, Mod.Hist, qstep = 0.01, method = "DIST", dist = "norm",
wet.day = FALSE, optim.method = "CG")
qm4.proj <- doQmap(Mod.Proj, qm4.fit)
qm4.hist <- doQmap(Mod.Hist, qm4.fit)


### QM5: Quantile delta mapping
library(MBC)
qm5.proj <- QDM(o.c = Obs, m.c = Mod.Hist, m.p = Mod.Proj, jitter.factor =
0.01)$mhat.p
qm5.hist <- QDM(o.c = Obs, m.c = Mod.Hist, m.p = Mod.Proj, jitter.factor =
0.01)$mhat.c
```

Figure S1. Monthly average minimum temperature in Atlanta (2050-2099) by climate model and quantile-mapping methods.

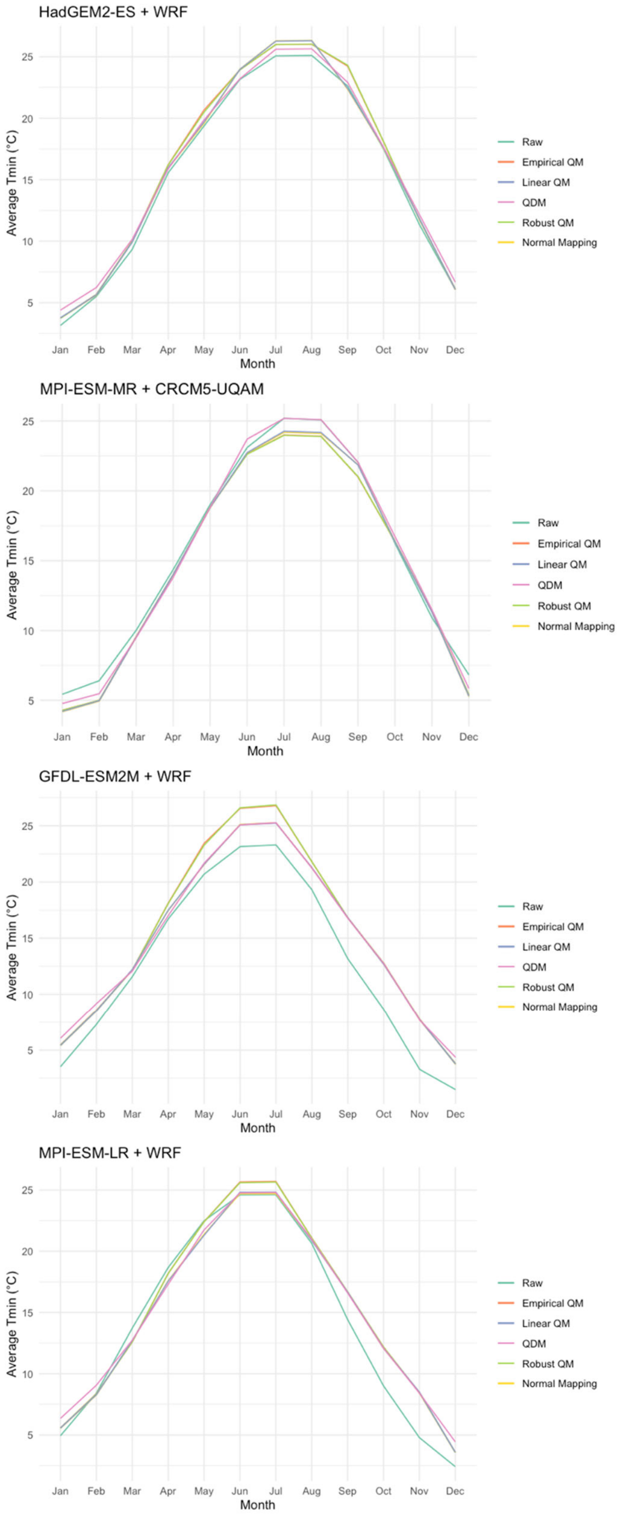

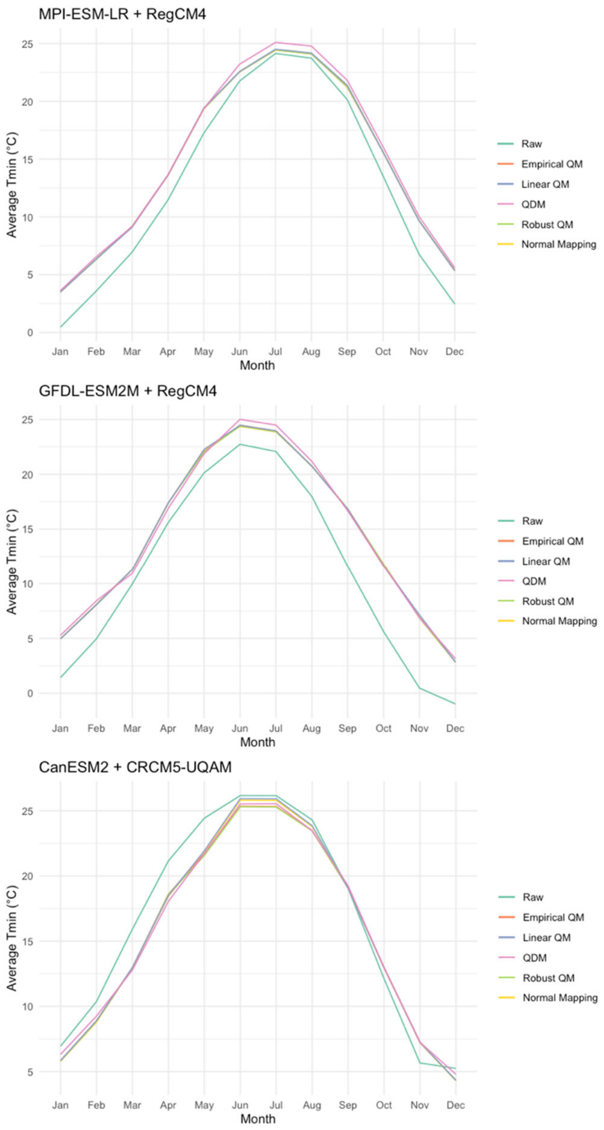
MPI-ESM-LR + RegCM4
Average Tmin (°C)
25
20
15
10
5
0
Jan
Feb
Mar
Apr
May
Jun
Jul
Aug
Sep
Oct
Nov
Dec
Month
Raw
Empirical QM
Linear QM
QDM
Robust QM
Normal Mapping
GFDL-ESM2M + RegCM4
Average Tmin (°C)
25
20
15
10
5
0
Jan
Feb
Mar
Apr
May
Jun
Jul
Aug
Sep
Oct
Nov
Dec
Month
Raw
Empirical QM
Linear QM
QDM
Robust QM
Normal Mapping
CanESM2 + CRCM5-UQAM
Average Tmin (°C)
25
20
15
10
5
Jan
Feb
Mar
Apr
May
Jun
Jul
Aug
Sep
Oct
Nov
Dec
Month
Raw
Empirical QM
Linear QM
QDM
Robust QM
Normal Mapping

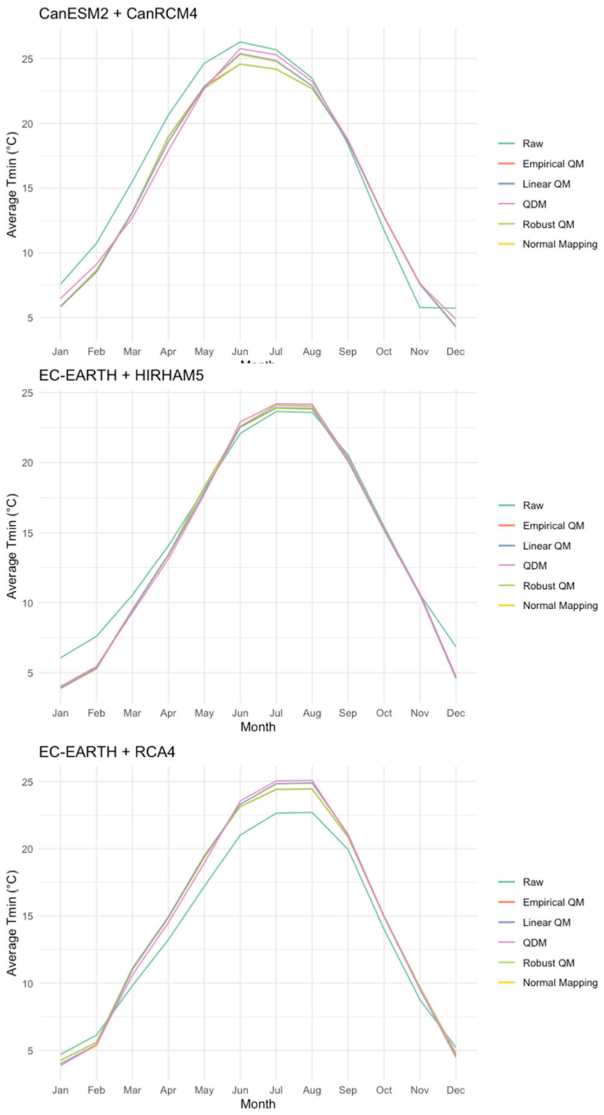
CanESM2 + CanRCM4
EC-EARTH + HIRHAM5
EC-EARTH + RCA4
Average Tmin (°C)
Month
Jan
Feb
Mar
Apr
May
Jun
Jul
Aug
Sep
Oct
Nov
Dec
5
10
15
20
25
Raw
Empirical QM
Linear QM
QDM
Robust QM
Normal Mapping
